# Prevalence of specific and recurrent/founder pathogenic variants in *BRCA* genes in breast and ovarian cancer in North Africa

**DOI:** 10.1186/s12885-022-09181-4

**Published:** 2022-02-25

**Authors:** Oubaida ElBiad, Abdelilah Laraqui, Fatima El Boukhrissi, Chaimaa Mounjid, Maryame Lamsisi, Tahar Bajjou, Hicham Elannaz, Amine Idriss Lahlou, Jaouad Kouach, Khadija Benchekroune, Mohammed Oukabli, Hafsa Chahdi, Moulay Mustapha Ennaji, Rachid Tanz, Yassir Sbitti, Mohammed Ichou, Khalid Ennibi, Bouabid Badaoui, Yassine Sekhsokh

**Affiliations:** 1 Laboratoire de Recherche et de Biosécurité P3, Hôpital Militaire d’Instruction Mohammed V, Rabat, Maroc; 2 Unité de séquençage, Laboratoire de Virologie, Centre de Virologie, des Maladies Infectieuses et Tropicales, Hôpital Militaire d’Instruction Mohammed V, Faculté de Médecine et de Pharmacie, Université Mohammed V, Rabat, Maroc; 3 Laboratoire de Biodiversité, Ecologie et Génome, Faculté des Sciences, Université Mohammed V, Rabat, Maroc; 4 Centre de virologie, des maladies infectieuses et tropicales, Hôpital militaire d’Instruction Mohammed V, Faculté de Médecine et de Pharmacie, Université Mohammed V, Rabat, Maroc; 5 Laboratoire de Biochimie-Toxicologie, Hôpital Militaire Moulay Ismail Meknès, Faculté de Médecine et de Pharmacie, Université Sidi Mohamed Ben Abdellah, Fès, Maroc; 6 Laboratoire de Virologie, Microbiologie, Qualité, Biotechnologies/Ecotoxicologie et Biodiversité, Faculté des sciences et techniques, Mohammadia, Université Hassan II, Casa, Maroc; 7 Service de Gynécologie Obstétrique, Hôpital militaire d’Instruction Mohammed V, Faculté de Médecine et de Pharmacie, Université Mohammed V, Rabat, Maroc; 8 Laboratoire d’Anatomopathologie, Hôpital militaire d’Instruction Mohammed V, Faculté de Médecine et de Pharmacie, Université Mohammed V, Rabat, Maroc; 9 Service d’Oncologie Médicale, Hôpital militaire d’Instruction Mohammed V, Faculté de Médecine et de Pharmacie, Université Mohammed V, Rabat, Maroc

**Keywords:** *BRCA* genes, Specific variant, recurrent/founder variant, North Africa

## Abstract

**Background:**

Elucidation of specific and recurrent/founder pathogenic variants (PVs) in *BRCA* (*BRCA1* and *BRCA2*) genes can make the genetic testing, for breast cancer (BC) and/or ovarian cancer (OC), affordable for developing nations.

**Methods:**

To establish the knowledge about *BRCA* PVs and to determine the prevalence of the specific and recurrent/founder variants in *BRCA* genes in BC and/or OC women in North Africa, a systematic review was conducted in Morocco, Algeria, and Tunisia.

**Results:**

Search of the databases yielded 25 relevant references, including eleven studies in Morocco, five in Algeria, and nine in Tunisia. Overall, 15 studies investigated both *BRCA1* and *BRCA2* genes, four studies examined the entire coding region of the *BRCA1* gene, and six studies in which the analysis was limited to a few *BRCA1* and/or *BRCA2* exons. Overall, 76 PVs (44 in *BRCA1* and32 in *BRCA2*) were identified in 196 BC and/or OC patients (129 *BRCA1* and 67 *BRCA2* carriers). Eighteen of the 76 (23.7%) PVs [10/44 (22.7%) in *BRCA1* and 8/32 (25%) in *BRCA2*] were reported for the first time and considered to be novel PVs. Among those identified as unlikely to be of North African origin, the *BRCA1* c.68_69del and *BRCA1* c.5266dupC Jewish founder alleles and PVs that have been reported as recurrent/founder variants in European populations (ex: *BRCA1* c.181T>G, *BRCA1* c1016dupA). The most well characterized PVs are four in *BRCA1* gene [c.211dupA (14.7%), c.798_799detTT (14%), c.5266dup (8.5%), c.5309G>T (7.8%), c.3279delC (4.7%)] and one in *BRCA2* [c.1310_1313detAAGA (38.9%)]. The c.211dupA and c.5309G>T PVs were identified as specific founder variants in Tunisia and Morocco, accounting for 35.2% (19/54) and 20.4% (10/49) of total established *BRCA1* PVs, respectively. c.798_799delTT variant was identified in 14% (18/129) of all *BRCA1* North African carriers, suggesting a founder allele. A broad spectrum of recurrent variants including *BRCA1* 3279delC, *BRCA1* c.5266dup and *BRCA2* c.1310_1313detAAGA was detected in 42 patients. *BRCA1* founder variants explain around 36.4% (47/129) of BC and outnumber *BRCA2* founder variants by a ratio of ≈3:1.

**Conclusions:**

Testing BC and/or OC patients for the panel of specific and recurrent/founder PVs might be the most cost-effective molecular diagnosis strategy.

## Introduction

Breast cancer (BC) became the most common cancer globally as of 2021, with an estimated 2.3 million new cases, representing 11.7% of all cancer cases [1]. According to the GLOBOCAN Cancer Tomorrow prediction tool, incident cases are expected to increase by more than 46% by 2040. The increasing global BC burden is mainly observed in low- and middle-income countries, particularly women under the age of fifty [2]. The rapid changes in diets and lifestyles, and social-cultural environments brought about by growing economies have had an impact on the prevalence of factors associated with increased BC risk. Therefore, the increase in the proportion of women in the industrial work force gave rise to a postponed pregnancy, having fewer children and, excessive total and abdominal body fat and physical inactivity which resulted in a convergence toward the risk factor profile of countries in Western Europe and narrowing international gaps in BC morbidity [1].

BC incidence rates increased uniformly and rapidly in transitioning countries. Some of the most rapid increases are occurring in North Africa, including Morocco, Algeria, Tunisia, Libya, and Mauritania. The incidence of BC among North African women aged 15 to 49 is lower than that in Western countries. However, the young age pyramid of North Africa makes the relative proportion of BC in young patients substantially higher than that in Western countries (e.g.50 to 60% versus 20% in France) [3]. The size and grade of breast tumors in North Africa is higher and the median age of onset (48) is more than ten years younger than the European/North American median of 61 [4], and is often diagnosed in premenopausal women. The relative frequency of triple-negative and inflammatory BC is also higher [3]. The combination of lower incidence and lower age of onset of BC in North Africa suggests that genetic factors such as germline pathogenic variants (PVs) in *BRCA* (*BRCA1* and *BRCA2*) genes may contribute to a larger proportion of BC overall.

Prevalence and PVs distribution of *BRCA* genes can vary in different regions and among different ethnic groups due to specific and recurrent/founder variants. Founder variants originated from an ancestor population and maintained over time, were observed in specific geographic areas [5]. Significant evidence from founder mutation has been described in Ashkenazi Jewish, Icelandic, French-Canadian, Brazilian and Italian populations [5]. Traditionally, well-documented founder *BRCA* PVs have been instrumental to informed prioritization strategies for time- and cost-effective genetic testing and prompt identification of carrier individuals [6]. Given the high rates of consanguinity and endogamy marriage culture among the North African populations, it seems plausible that specific and recurrent/founder *BRCA* PVs may be detected in the region. Rebbeck et *al*. investigated 1650 and 1731 unique PVs in *BRCA1* and *BRCA2* genes, respectively, from 29,700 families worldwide and observed distinct variation in PV type or prevalence by geographical region and race/ethnicity [7]. Racial and ethnic differences can play an important role in hereditary breast carcinomas through its associations with specific and recurrent/founder variants. The purpose of this review is to establish the knowledge about *BRCA* PVs and to determine the prevalence of the specific and recurrent/founder variants in *BRCA* genes in BC and/or OC women in Morocco, Algeria and Tunisia. It seems that no surveys on *BRCA* PVs have yet been conducted in Libya and Mauritania, therefore no data are available.

## Methods

We conducted a systematic review of all the literature published on the *BRCA* PVs spectrum and the frequencies in BC women in North Africa. Pub Med, Science Direct and Google Scholar were searched up to June 2021 for eligible studies using the following keywords: “breast cancer”, or “breast tumor”, or “adenocarcinoma of the breast”, or “*BRCA* genes”, or “*BRCA1* gene”, or “*BRCA2* gene”, or “*BRCA* pathogenic variant”, or “*BRCA* mutation”, or “*BRCA* prevalence” or “*BRCA* frequency”, or “*BRCA* rate” or “*BRCA* incidence”. An additional literature search was also conducted using North Africa and specific country names belonging to the considered region and any other variant names for any of North Africa countries (ex: Mediterranean countries, Maghreb, Arab population). We manually checked reference lists of the included studies and relevant reviews to identify additional studies. We also searched relevant abstracts reported in the most important multi-disciplinary societies of medical oncology such as the American Society of Clinical Oncology (ASCO) meetings to identify unpublished studies.

Original research articles were identified from Morocco, Algeria, and Tunisia. The included studies had to meet the following criteria: the study must relate to the role of *BRCA1* and/or *BRCA2* genes in BC and/or OC, it should also analyze all the coding regions, test for known mutations, or select exons of *BRCA* genes. Besides, the study must provide sufficient information on the *BRCA1* and/or *BRCA2* PV frequencies. Likely pathogenic variants or variants of unknown/uncertain significance (VUS) were excluded from this study. The prevalence of any variant was included regardless of whether the variant was specific or recurrent/founder. Also, where study authors did not clearly state that variant was germline or somatic and/or pathogenic, or clinically relevant, the variant was classified as not reported/unclear in order to avoid any misinterpretation. Details of the study methods, population characteristics, and prevalence of *BRCA* PVs were extracted and summarized in Table 1.

**Table 1:** Details of studies examining *BRCA1* and *BRCA2* genes in North Africa

	Number of patients	*BRCA* carriers	Mean age	Methods	Covered gene region
**Morocco**					
Laarabi et al. (2011) [8]	8	6	NA	Direct sequencing	All
Tazzite et al. (2012) [9]	40	10	38	Direct sequencing	All
Laraqui et al. (2013) [10]	121	7	44	Direct sequencing	*BRCA1*
Elkhachibi et al. (2015) [11]	71	2	41	HRM, Direct sequencing	Exon11(*BRCA1*)
Jouali et al. (2016) [12]	15	6	47	NGS	All
Quiles et al. (2016) [13]	11	8	36.5	Direct sequencing	Exon20 (*BRCA1*)
Laarabi et al. (2017) [14]	122	14		Direct sequencing (51 patients), NGS (23 patients), Target screening (*BRCA2* exon 10,48 patients)	All, Exon10 (*BRCA2*)
El Ansari et al. (2020) [15]	64	18	42	NGS	All
Bakkach et al. (2020) [16]	33	4	35	NGS	All
Mansouri et al (2020) [17]	32	7	45	NGS	All
Jouali et al. (2020) [18]	39	1	46	NGS	All
**Algeria**					
Uhrhammeret al. (2008) [19]	51	5	31.5	Direct sequencing, MLPA	*BRCA1*
Cherbal et al. (2010) [20]	86	10	NA	Direct sequencing, MLPA	All
Henouda et al. (2016) [21]	40	8	36.6	Direct sequencing, MLPA	All
Boulenouar et al. (2018) [22]	50	4	NA	Direct sequencing, MLPA	All
Mehemmai et al. (2019) [23]	113	7	44	Direct sequencing, NGS	Exon3, 4,and 10 (*BRCA1*), Exon10 (*BRCA2*)
**Tunisia**					
Troudi et al. (2007) [24]	36	7	56.8	Direct sequencing	All
Troudi et al. (2008) [25]	32	5	46.5	Direct sequencing	*BRCA1*
Mahfoudh et al. (2011) [26]	24	6	41	Direct sequencing	*BRCA1*
Riahi et al. (2013) [27]	48	12	NA	Direct sequencing	All
Fourati et al. (2014) [28]	66	12	45	Direct sequencing	*BRCA1* (5,20, and part of 11), *BRCA2* (10,11)
Msolly et al. (2015) [29]	17	1	45.8	Direct sequencing	All
Mahfoudh et al. (2019) [30]	33	2	53.8	Direct sequencing	All
Mighri et al. (2020) [31]	112	9	NA	Direct sequencing, NGS	All
Guerfali et al. (2021) [32]	134	19	NA	NGS	All

HRM: High Resolution Melt, MLPA: Multiplex Ligation-dependent Probe Amplification, NGS: Next generation sequencing, NA: not available

A reinterpretation of sequence variants was conducted by following the classification system recommended by the American College of Medical Genetics and Genomics-Association for Molecular Pathology (ACMG-AMP) Standards and Guideline for the Interpretation of Sequence Variants [33]. The 2015 ACMG-AMP guidelines were a major step toward establishing a common framework for variant classification. The ACMG-AMP suggests that the clinical pathogenicity of a variant can be evaluated using multiple lines of evidence from available literature, structural/functional data, population frequencies, and statistical analyses of clinical data. The process can result in 1 of 5 classifications: benign (class 1), likely benign (2), VUS (class 3), likely pathogenic (class 4), and pathogenic (class 5).

Variants were considered to be “founder” if they were described as such in the primary literature, based on confirmatory haplotype analysis or population frequency.

## Results

The search of the databases yielded 25 relevant references which are closely related to defining the inclusion criteria and was included in this review. The retrieved articles describe studies conducted in Morocco (n=11) [8–18], Algeria (n=5) [19–23], and Tunisia (n=9) [24–32]. Overall, 15 studies investigated both *BRCA1* and *BRCA2* genes [8, 9, 12, 15–18, 20–22, 25–27, 29, 31, 32], four studies examined the entire coding regions of the *BRCA1* gene [10, 19, 24, 30], and six studies in which the analysis was restricted to a few *BRCA1* and/or *BRCA2* exons [11, 13, 14, 23, 28].

Overall, we observed 76 distinct *BRCA* PVs (44 in *BRCA1* and 32 in *BRCA2*). The identified variants are found in 196 BC and/or OC patients (129 *BRCA1* carriers and 67 *BRCA2* carriers). A total of 18 of the 76 (23.7%) PVs [10/44 (22.7%) in *BRCA1*, 8/32 (25%) in *BRCA2*] were reported for the first time and were considered to be novel PVs in the North African populations. Among them, four PVs were reported in Morocco (c.3453delT in *BRCA1* [12], and c.3381delT, c.7110delA, c.7234_7235insG in *BRCA2* [9], three in Algeria (deletion of exon2 in *BRCA1* [20], c.2805delAand c.6450del in *BRCA2* [21], and eleven in Tunisia (c.211dupA [24, 25], c.296_297delTG [32], c.2418dupA [31], c.3254delG [32], c.3364_3370dupACAGATT [32], c.3751dup [28], c.4067_4071delAAGAA in *BRCA1* [32] and c.1313dupT [27], c.1976_1800delCTTAT [27], c.2095 C>T [32], c.7654dupT [27] in *BRCA2*). The reported PVs in *BRCA* genes from North African studies are presented in Table 2.

**Table 2:** Pathogenic *BRCA1* and *BRCA2* variants identified in North African breast/ovarian cancer patients

	Exon	Mutation type	Protein consequence	Number of cases	Country	Cancer site	Familial Sporadic	References
***BRCA1*** **Pathogenic variants**								
c.46_74del29	2	FS	p.Asn16fs	2	Algeria	BC	1 Familial 1 Sporadic	[19]
c.66_67delAG	2	FS	p.Glu23fs	2	Morocco	BC	Familial	[15]
c.68_69delAG	2	FS	p.Glu23fs	4	Morocco	3 BC, 1 OC	Familial	[8]
Del exon 2	2	LGR	-	2	Algeria	1 BOC, BC	Familial	[20, 21]
c.83_84delTG	3	FS	p.Arg28fs	2	Algeria	BC	1 Familial 1 Sporadic	[19, 20]
c.116G>A	3	MS	p.Cys39Tyr	1	Morocco	BC	Familial	[17]
c.181T>G	5	MS	p.Cys61Gly	3	Morocco, Algeria	BC	Familial	[12, 33]
c.211dupA	5	FS	p.Arg71fs	19	Tunisia	3 BC, 2 BOC	Familial	[24, 25, 27, 28, 31]
c.212+2insG	5	Splicing	-	1	Tunisia	BC	Familial	[26]
Del exon 8	8	LGR	-	1	Algeria	BC	Familial	[20]
c.2338C>T	10	NS	p.Gln780Ter	2	Tunisia	OC	1 Familial 1 Sporadic	[32]
c.798_799delTT	11	FS	p.Ser267LysfsX19	18	Morocco, Algeria, Tunisia	BC	16 Familial 2 Sporadic	[9, 10, 15–20, 26, 29]
c.1016dupA	11	FS	p.Val340LysfsX6	3	Morocco	BC	Familial	[10, 15]
c.1504_1508delTTAAA	11	FS	p.Leu502fs	1	Tunisia	BC	Familial	[27]
c.1817delC	11	FS	p Pro606Leufs6	2	Algeria	BC	1 Familial 1 Sporadic	[19, 21]
c.202+1G>A	11	FS	-	1	Algeria	BC	Familial	[19]
c.296_297delTG	11	FS	p.V99fs*9	1	Tunisia	BC	Familial	[32]
c.2125_2126insA	11	FS	p.Phe709Tyrfs	4	Morocco, Algeria	BC	3 Familials 1 Sporadic	[12, 16, 17, 22]
c.2418dupA	11	MS	p.Ala807Serfs	1	Tunisia	BC	Familial	[31]
c.2551delG	11	FS	p.Glu851fs	2	Tunisia	BC	Familial	[24, 25]
c.2745dupT	11	NS	p.Ser915fs	1	Algeria	BC	Sporadic	[19]
c.2805delA	11	FS	p.Asp936Ilefs	2	Morocco	BC	Familial	[9, 11]
c.3254delG	11	FS	p.Arg1085Asnfs2	1	Tunisia	BC	Familial	[32]
c.3279delC	11	FS	p.Tyr1094Ilefs	6	Morocco	BC	Familial	[9, 11, 15]
c.3331_3334delCAAG	11	FS	p.Gln1111fs	1	Tunisia	BC	Familial	[26]
c.3364_3370dupACAGATT	11	FS	Stop1115	1	Tunisia	BC	Familial	[32]
c.3453delT	11	FS	p.Asp1151Glufs	1	Morocco	BC	Familial	[12]
c.3715delT	11	FS	p.Ser1239fs	1	Algeria	BC	Sporadic	[19]
c.3751dup	11	FS	p.Thr1251fs	1	Tunisia	OC	Familial	[28]
c.4041_4042delAG	11	FS	p.Gly1348fs	4	Tunisia	1 BC, 2 BOC, 1MBC	Familial	[24, 25, 28, 32]
c.4065_4068del	11	FS	p.Asn1355Lysfs10	1	Algeria	BC	Familial	[21]
c.4067_4071delAAGAA	11	FS	p.GLn1356Argfs8	1	Tunisia	BC	Familial	[32]
c.5095C>T	14	MS	p. Arg1699Trp	2	Morocco	BC	Familial	[10]
c.4676- ? _4986 + ? Del/p. ?	15	?	-	2	Algeria	1 BOC,1 BC	Familial	[23]
c.5030_5033delCTAA	15	NS	p.Thr1677Ilefs2	3	Tunisia	1 BOC,2 OC	Familial	[32]
c.4823C>G	16	NS	p.Ser1608Ter	1	Morocco	BC	Familial	[15]
c.4942A>T	16	NS	p.Lys1648X	1	Morocco	BC	Sporadic	[10]
c.5062_5064delGTT	17	FS	p.Val1688del	1	Morocco	BC	Familial	[9]
c.5117G>C	18	MS	p.Gly1706Ala	1	Algeria	BC	Familial	[22]
c.5158C>T	18	MS	p.Arg1720Trp	1	Morocco	BC	Familial	[15]
c.5266dupC	20	FS	p.Gln1756Profs	11	Tunisia	9 BC, 2 BOC	Familial	[24–28, 30]
c.5309G>T	20	MS	p.Gly1770Val	10	Morocco	9BC,1 OC,	8 Familial 2 Sporadic	[13, 17]
c.5332+1G>A	20	Splicing	-	2	Algeria	1 BC, 1 OC	Familial	[21, 23]
c.5390C>A	22	NS	p.Ser1797Ter	1	Morocco	BC	Familial	[17]
***BRCA2*** **Pathogenic variants**								
c.17_20delAAGA	2	FS	p.Lys6Argfs17	2	Tunisia	BC	Familial	[32]
c.250C>T	3	NS	p.Gln84Ter	1	Algeria	BC	Familial	[32]
c.289G>T	3	NS	p.Glu97Ter	1	Morocco	BC	Familial	[22]
c.517_1G>A	Intron 6	SA	-	1	Morocco	BC	Sporadic	[9]
c.632_1G>A	7	Splicing	-	2	Tunisia	BC	Familial	[32]
c.1302_1305delAAGA	10	FS	p.Lys437fs	1	Morocco	OC	Familial	[15]
c.1309del4	10	FS	Stop459	1	Tunisia	BC	Familial	[24]
c.1310_1313delAAGA	10	FS	p.Lys437IlefsX22	26	Morocco, Algeria, Tunisia	25 BC, 1MBC	Familial	[12, 14, 20, 28, 31]
c.1313dupT	10	FS	Stop451	2	Tunisia	BC	Familial	[27]
c.1528G>T	10	NS	p.Glu510	1	Algeria	BC	Familial	[21]
c.1813dupA	10	FS	p. Ile605Asnfs11	1	Algeria	BC	Familial	[23]
c.1976_1800delCTTAT	10	FS	p.Ser599	1	Tunisia	BC	Familial	[32]
c.2095C>T	10	NS	p.Gln699Ter	1	Tunisia	BC	Familial	[32]
c.5116_5119delAATA	11	FS	p.Arg2108Cys	2	Morocco	BC	Familial	[16, 17]
c.3381delT	11	FS	p.Phe1127Leufs	1	Morocco	BC	Familial	[16]
c.3847_3848delGT	11	FS	p.Val1283fs	1	Morocco	OC	Familial	[9]
c.3860delA	11	FS	p.Asn1287fs	1	Morocco	BOC	familial	[15]
c.5073dupA	11	FS	p.Trp1692Metfs	2	Morocco	BC	Familial	[8]
c.5576_5579delTTAA	11	FS	p.I1859fs	1	Morocco	OC	familial	[15]
c.5682insA	11	FS	-	1	Tunisia	BC	familial	[24]
c.5722_5723delCT	11	FS	p.Leu1908Argfs2	1	Algeria	BC	familial	[20]
c.6450del	11	FS	p.Val2151Phefs17	1	Algeria	BC	familial	[21]
c.7110delA	14	FS	p.Lys2370Asnfs	2	Morocco	1 OC, 1BC	familial	[9, 15]
c.7234_7235insG	14	FS	p.Thr2412fs	3	Morocco	BC	Familial	[9, 12]
c.7235_7236insG	14	FS	p.Lys2413fs	1	Morocco	OC	Familial	[15]
c.7654dupA	16	FS	p.Ile2552Asnfs2	2	Algeria	BC	Familial	[21, 23]
c.7654dupT	16	FS	p.Ile2552fs	2	Tunisia	BC	Familial	[27]
c.8485C>T	19	NS	p.Gln2829	1	Algeria	BC	familial	[23]
Del exons 19-20	19/20	LGR	-	1	Algeria	BC	familial	[21]
c.8940delA	22	FS	p.Glu2981Lysfs7	1	Algeria	BOC	Familial	[23]
c.9097delA	22	FS	p.Thr3033Leufs	1	Tunisia	BC	Familial	[32]
c.9364G>A	25	MS	p.Ala3122Thr	1	Algeria	BC	familial	[22]

FS: frameshift mutation, LGR: Large genomic rearrangement, MS: missense mutation, BC: breast cancer, OC: ovarian cancer, BOC: breast and ovarian cancer, NS: Nonsense mutation

Among those identified PVs, some are unlikely to be of North African origin which includes the *BRCA1* c.68_69del and *BRCA1* c.5266dupC Jewish founder variants, as well as PVs that have been reported as founder variants in European populations (ex: *BRCA1* c.181T>G in Poland). Furthermore, other PVs have been described worldwide and represented as common PVs in several populations (ex: *BRCA1* c.1016dupA in Italy, Germany, Scandinavian countries and French-Canadians, *BRCA1* c.2125_2126insA in French-Canadians, *BRCA1* c.2338 C>T and *BRCA2* c.1813dupA in Germany, *BRCA1* c.5030_5033delCTAA in France, *BRCA2* c.3860delA in Austrian population, and *BRCA2* c.3847_3848delGT in Denmark).

The most well characterized five PVs are four in *BRCA1* gene including c.211dupA (14.7%, 19/129) [24, 25, 27, 28, 31], c.798_799detTT (14%, 18/129) [9, 10, 15–18, 20, 24, 29, 30], c.5266dup (8.5%, 11/129) [24–28, 30], c.5309G>T (7.8%, 10/129) [13, 17], c.3279delC (4.7%, 6/129) [9, 11, 15] and one in *BRCA2* including c.1310_1313detAAGA (38.9%, 26/67) [12, 14, 20, 28, 32]. The *BRCA1* c.798_799delTT was identified in 18 North African patients, accounting for 14% (18/129) of the total identified *BRCA1* PVs [9, 10, 15–20, 24, 29, 30]. Microsatellite markers in and flanking the *BRCA1* locus showed a common haplotype in Algerian and Tunisian c.798_799delTT carriers, suggesting the first non-Jewish founder variant to be described in Northern Africa [19]. The c.798_799delTT variant, located in exon 11, is a frame-shift variant including two small deletions, two bases (TT) deletion, that cause truncated protein signal at codon 285.

The other frequently recurrent PVs c.211dupA [24, 25, 27, 28, 31] and c.5266dupC [24–28, 30] were found in 55.6% (30/54) of *BRCA1*-related hereditary breast and ovarian cancer (HBOC) in Tunisian families but neither in Algerian nor in Moroccan families with BC and/or OC. The c.211dupA variant seems to be the most frequent *BRCA* PVs in Tunisia, accounting for 35.2% (19/54) of all identified *BRCA1* PVs [24, 25, 27, 28, 31]. It seems to be specific to Tunisia since it has never been previously described in any other population. The haplotype analysis supported the founder effect of c.211dupA in Tunisia and showed its recent origin. The frameshift variant c.211dupA results in a premature protein termination at codon 79 at the level of the splicing donor site of exon 5.

The c.5266dupC variant in *BRCA1* exon 20 was detected in most Tunisian series, accounting for 20.4% (11/54) of all *BRCA1* PVs [24–27, 30]. This alteration is one of three well-characterized Ashkenazi Jewish founder mutations, with an overall carrier frequency of nearly 0.5% in this population. The c.5266dupC variant is the most globally frequent, pathogenic *BRCA1* variant and has been reported in varied populations in Africa, America, Asia and Europe. The haplotype analysis indicates the likelihood of a single founder origin both in Europe and in North America for the c.5266dupC variant [34]. Haplotype analysis may be useful in establishing whether or not it has a common founder origin for all *BRCA1* c.5266dup variant in Tunisia. The *BRCA1* c.5266dupC PV, located in coding exon 18, results from a duplication of C at nucleotide position 5266, causing a translational frameshift with a predicted alternate stop codon.

The *BRCA1* c.5309G>T variant was identified in ten patients of which nine of which were BC and one was OC. Two of the BC patients were sporadic cases [13, 17]. A haplotype linked to c.5309G>T, constructed from five microsatellite markers and spanning 1.54 Mb, was defined in one family. The alleles found in the other families are consistent with this haplotype, supporting the founder effect of c.5309G>T in Morocco [13, 35, 36]. This mutation was first reported in Spain in two families of Moroccan origin and was classified as probably pathogenic on the basis of a combination of functional and structural analyses [17]. The c.5309G>T variant in *BRCA1* gene is located in the functionally important *BRCA1* carboxyl-terminal domain, a domain known to harbor missense substitutions associated with increased risk of BC and/or OC. The c.5309G>T variant should be treated as a disease-causing variant despite a lack of evolutionary conservation of the glycine at position 1770 [35].

The *BRCA1* c.3279delC variant mutation is a recurring PV in the Moroccan population. It accounts for 12.2% (6/49) of the *BRCA1* mutations [9, 11, 15], suggesting a possible founder effect. This mutation has been identified in one HBOC kindred of Dutch descent as well as in one HBOC kindred of Moroccan descent in which the proband and her mother were diagnosed with BC at younger ages [9, 37]. The c.3279delC variant, located in coding exon 9 of the BRCA1 gene, is a frameshift variant of one base (C) deletion that changes a tyrosine to isoleucine at codon 1094 and creates a premature stop codon at position 15 of the new reading frame.

The *BRCA2* c.1310_1313delAAGA frameshift PV is considered as a North African recurrent mutation since it has been identified in Moroccan [12, 14], Algerian [20], and Tunisian [28] BC patients. Interestingly, geographical clustering in the North-Eastern area of Morocco is evident for the c.1310_1313delAAGA mutation, suggesting a founder effect [14]. c.1310_1313delAAGA incidence rate is high and accounts for 50% (17/34) of all *BRCA2* PVs in North-East of Morocco [14]. This sequence change harbors the deletion of four nucleotides from exon 10 of the *BRCA2* mRNA, causing a frameshift after codon 437 and the creation of a premature translational stop signal 22 amino acid residues.

## Discussion

*BRCA* genes remain the primary inherited causes of BC and OC, accounting for 30–70% of hereditary BC families and approximately 90% of hereditary OC families. *BRCA* PV carriers are linked with an increased lifetime risk of developing BC and/or OC. Current investigations have reported several differences and significant heterogeneity in the incidence and geographic distribution of PVs in *BRCA* genes [7, 38]. In North Africa, *BRCA* PVs frequency varies widely from ≈1% (Morocco) in sporadic BC [10] to 37.5% (Tunisia) in HBOC [24]. The spectrum and prevalence of *BRCA* PVs vary mainly due to population-specific recurrent/founder variants. Some of which have documented a founder effect of such recurrent or unique variants through haplotype analysis. Prevalence studies of *BRCA* gene variants suggest that these genetic alterations can explain a high-frequency BC in some populations than others and may contribute to differences in cancer risk between populations and racial/ethnic minorities [5–7]. Accurate identification of the population-specific variant spectrum is therefore the first step towards incorporating appropriate *BRCA* genetic testing into clinical practice in certain populations and racial/ethnic groups [39].

In North Africa, *BRCA1* founder variants explain around 36.4% (47/129) of BC and outnumber *BRCA2* founder variants by a ratio of ≈3:1. Clear founder effects have been reported in Morocco (*BRCA1* c.5309G>T) and Tunisia (*BRCA1* c.211dupA). Furthermore, the *BRCA1* c.798_799detTT was identified in 14% (18/129) of *BRCA1* carriers in North African populations. It was initially thought to be specific to Algeria (19.23%, 5/26) [19, 20], but later found to be prevalent in Tunisia (7.4%, 4/54) [24, 29, 30] and Morocco (18.4%, 9/49) [9, 10, 15–18]. Haplotype analysis of some families carrying these PV revealed the presence of a common allele [19]. The *BRCA1* c.798_799detTT frameshift variant is cited twice in the Breast Cancer Information Core (BIC) database, without any ethnic origin indicated. Interestingly, the c.798_799delTT and c.5309G>T variants in *BRCA1* have been identified in sporadic BC patients in North Africa, and hence their presence in patients without a history of BC and/or OC cannot be attributed to the de novo mutational event. To our knowledge, the *BRCA1* c.798_799delTT variant has been identified in Spain [40], in southern Italy [41, 42], and in France [43]. This restricted geographical distribution to close Mediterranean countries could be explained by geographical proximity and migration flow history. The c.1310_1313delAAGA variant in *BRCA2* represents another common PV in North African populations whose founder effect through haplotype analysis is required for confirmation in this region. According to the BIC database, the *BRCA2* c.1310_1313delAAGA PV was found in different European patients and was recorded several times in the French Universal Mutation database-*BRCA2* (UMD-*BRCA2*) and classified as founder variant [44]. The c.1310_1313delAAGA PV, located in coding exon 9 of the *BRCA2* gene, results from a deletion of four nucleotides at nucleotide positions 1310 to 1313. The deletion causes a frameshift, which changes a Lysine to an Isoleucine at codon 437, and creates a premature stop codon at position 22 of the new reading frame. In addition, it is important to highlight that one of the three Ashkenazi Jewish founder variants (i.e. *BRCA1* c.5266dup) is frequently observed in Tunisian *BRCA1* carriers (20.4%, 11/54). The *BRCA1* c.5266dup has been mainly observed in Italy in some families from the North-eastern coast of Sicily [45]. *BRCA1* c.5266dup was reported as a founder variant also in several European populations with a low proportion of individuals who self-identify as Jewish [46]. Previous studies indicate that the mutation was introduced into the Ashkenazi Jewish genetic pool approximately 400-500 years ago in Poland, but the mutation originated from a single common European ancestor long before it became an Ashkenazi Jewish founder mutation [47]. The *BRCA1* c.5266dupC variant is the second most frequently reported PV in the BIC database.

Besides recurrent/founder variants, two *BRCA1* PVs (c.211dupA and c.5309G>T) identified in North Africa were not reported previously. The c.211dupA and c.5309G>T variants in the *BRCA1* gene were found as unique to the Tunisian and Moroccan populations, respectively [24, 25, 27, 28, 31]. Several founder variants as they have common ancestral haplotypes have been identified in various areas and races. For example, the well-known founder variants are c.68_69delAG, c.5266dupC in *BRCA1*, and c.5946_5946delT in *BRCA2* in the Ashkenazi Jewish population [48, 49]. The c.824_825ins10, c.1713_1717delAGAAT, c.5177_5180del4, c.4357+1G>A variants in *BRCA1* and c.4471_4474delCTGA variant in *BRCA2* have been identified as a potential Afro-American founder variant [50–52]. A high frequency of founder c.771_775del5 *BRCA2* PV was identified in Iceland [53]. The founder *BRCA2* c.771_775del5 variant was reported to cause the familial clustering of both female and male BC cases [53, 54]. The c.2685_2686del variant in *BRCA1* and c.9672dup variant in *BRCA2* have been reported as founder mutations for the Dutch population [55]. A French-Canadian founder status is evident for c.4327 C>T in *BRCA1* and c.8537_8538delAG in *BRCA2* [56]. The founder effect of c.68_69delAG, c.181T>G, c.676delT, c.1687 C>T, c.3700_3704delGTAAA, c.3756_3759delGTCT, c.4035delA, c.5251 C>T, c.5266dupC in *BRCA1* and c.658_659delGT, c.3847_3848delGT, c.5946delT, c.7913_7917delTTCCT in *BRCA2* is characteristic of Central European population [46]. In *BRCA1* gene, three large genomic deletions (deletion of exon 20, exon 24, and exons 23, and 24) and the c.5212G>A PV have been characterized as population-specific founder variants by haplotype analysis in the Greek population [57]. The c.1140dupG and c.4136_4137delCT variants in *BRCA1* were identified as novel putative founder variants in Middle Eastern patients [58]. In Addition to c.2641G>T, c.68_69delAG, c.5266dupC, and c.1374delC recurrent variants in *BRCA1*, haplotype analysis confirmed the founder status of c.5771_5774del and c.7934del variants in *BRCA2* and revealed an additional founder variant in *BRCA2*, c.582G>A, in South African families [59]. In Latin America, clear founder effects have been reported in Mexico (*BRCA1* del exons 9–12), Brazil (*BRCA1* c.5266dupC and *BRCA2* c.156_157insAlu), and Colombia (*BRCA1* c.3331_3334delCAAG, *BRCA1* c.5123 C>A, and *BRCA2* c.2808_2811delACAA) [60]. In the Middle Eastern population, nine PVs were recurrent in epithelial OC and founder mutation analysis revealed only two mutations (*BRCA1* c.4136_4137delCT and *BRCA1* c.1140dupG) sharing the same haplotypes thus representing founder mutations [61]. Studying the founder effect of these variants can provide a comprehensive analysis of a population’s evolution and its migration pathways.

The identification of recurrent/founder variants is an extremely important step towards the improvement of genetic counseling since molecular testing can be targeted to the recurrent/founder variant allowing for a more rapid and less expensive test [30]. The high frequency of recurrent/founder variants, allowing for analyzing a large number of cases, might provide accurate information regarding their penetrance and distinguish factors that affect them. Once a risk factor is identified in one subgroup of PV carriers it would need to be tested through other PV carriers. Subsequently, it would need to be tested in a large population-based case-control study of patients with BC and/or OC, in order to determine how important the risk factor is in the general population [62]. Furthermore, the evidence of differences in susceptibility and in age onset of cancer and in the type of cancers that develop among carriers of a founder variant could make it possible to define the role and importance of risk-modifying factors with the resulting improved disease management [5].

Furthermore, a specific variant in *BRCA* genes has been found with high prevalence in restricted populations as a consequence of a founder effect. The specific founder variant of c.303T>G and c.2641G>T in *BRCA1* gene were reported in the Yoruba population from Nigeria [63] and in the Afrikaner population from South Africa [59], respectively. Geographical clustering of c.3481_3491del11 and c.5128G>T variants in *BRCA1* in the Alsace-Lorraine region at the North-East and in the north-east of France, respectively, suggests a founder effect [43, 64]. In the southwest of the Netherland, the founder effect of c.4186-1643_4357+2020del3835 variant in *BRCA1* gene and c.5351dupA variant in *BRCA2* gene were prevalent in Catholic (West Brabant clustering) and Protestant (South Beveland clustering) families, respectively, reflecting religious endogamy [65]. The c.4964_4982del19 variant in *BRCA1* gene has been identified as a founder variant in a geographically and historically homogeneous population from Calabria, a south Italian region [66]. Significant regional founder effect has been demonstrated for c.3228_3229delAG, c.3285delA, c.1380dupA, and c.5062_5064del3 variants in *BRCA1* gene in Tuscany in central Italy [67]. The c.5062_5064delGTT variant in *BRCA1* gene and c.8537_8538delAG variant in *BRCA2* gene have been described as founder variants in Middle Sardinia and in South and Middle Sardinia, respectively [68]. Figlioli et *al*. showed that the *BRCA1* c.190T>C is a founder variant in BC families from Bergamo province in the Northern Italian region [6]. The c.3048_3052dupTGAGA variant is the western Swedish *BRCA1* founder variant [69]. The *BRCA1* c.131G PV was considered a specific founder variant in the Lebanese population [70]. In Saudi Arabia, six PVs were reported to be unique and founder PVs in Saudis with OC; four in *BRCA1* gene (c.711_712insTGAA, c.1140dupG, c.5054 C>T, c.5530delC) and two in *BRCA2* gene (c.2667delT and c.5760_5770del11) genes [71].

The identification of specific *BRCA* variants could allow us to identify new founder effects for some of these and to quantify the degree of homogeneity within a population. Moreover, it was essential for promoting and potentially advancing rapid founder-based *BRCA* point-of-care technology as a time- and cost-effective alternative. This discovery can surely help oncologists and cancer genetics professionals to simplify their choices in the genetic screening on high-risk families, on the basis of their ethnic origin, through more accurate estimation of carrier probabilities of *BRCA* variants. Understanding the contribution of specific variants to BC risk in such a population will help to examine the possibility of conducting population-wide genetic testing for candidate variants that are over-represented in this population [72]. The most well-known and significant examples of recurrent/founder mutations in *BRCA* genes found worldwide are presented in Table 3.

**Table 3:** Examples of recurrent and founder mutations in *BRCA1* and *BRCA2* genes described in European and non-European countries

	*BRCA1*		*BRCA2*	
	HGVS nomenclature	Effect on amino-acid	HGVS nomenclature	Effect on amino-acid
**African Countries by Population**				
Algerian [19]	c.798_799delTT*	p.Ser267Lysfs*		
Egyptian [73]	c.68_69delAG* c.181T>G c.4327C>T c.5266dupC c.5335delC*	p.Glu23fs* p.Cys61Gly p.Arg1443Ter p.Gln1756Profs p.Gln1779Asnfs*	c.771_775del5* c.5335del	p.Asn257Lysfs* p.Gln1779Asnfs
Moroccan [9, 11, 13–15, 19]	c.798_799delTT* c.3279delC c.5309G>T*	p.Ser267Lysfs* p.Tyr1094Ilefs p.Gly1770Val*	c.1310_1313detAAGA	p.Lys437Ilefs
Nigerian [74]	c.191G>A c.303T>G * c.1504_1508delTTAAA c.1623dupG* c.3268C>T c.4122_4123delTG* c.4240dupC c.5324T>G*	p.Cys64Tyr p.Tyr101Ter* p.Leu502Alafs p.Asn542fs* p.Gln1090Ter p.Ser1374Argfs* p.Leu1414fs p.Met1775Arg*	c.1310_1313delAAGA c.2402_2412del11 c.8817_8820delGAAA	p.Lys437Ilefs p.Asn801fs p.Lys2939fs
Senegalese [75]	c.815_824dup10*	p.Thr276Afs*		
South African [59]	c.68_69delAG c.1374delC c.2641G>T* c.5266dupC c.7934delG*	p.Glu23fs p.Asp458Glufs p.Glu881Ter* p.Gln1756Profs p.Arg2645Asnfs*		
Tunisian [19, 28, 30, 31]	c.211dupA* c.798_799delTT* c.5266dupC	p.Arg71Lysfs p.Ser267Lysfs p.Gln1756Profs	c.1310_1313detAAGA	p.Lys437Ilefs
**American countries by population**				
African American [50–52]	c.824_825ins10* c.1713_1717delAGAAT* c.4357+1G>A c.498616T>C c.5177_5180delGAAA* c.5251C>T c.5324T>G c.5387C>A c.4485-1G>A*	- p.Glu572fs* - - p.Arg1726Lysfs* p.Arg1751Ter p.Met1775Arg p.Ser1796Ter -	c.4471_4474delCTGA	p.Leu1491Lysfs
Argentinian [76]	c.68_69delAG* c.211A > G* c.5266dupC*	p.Glu23fs* p.Arg71Gly* p.Gln1756Profs*	c.2808_2811del4 c.5946_5946delT*	p.Ala938Profs p.Ser1982Argfs*
Bahamian [77–80]	c.68_69delAG* c.824_825ins10* c.4357+1G>A* c.4611_4612insG* c.49861+6T>C* c.5324T>G*	p.Glu23fs - - p.Gln1538fs - p.Met1775Arg	c.7900delA*	p.Met2634fsX14
Brazilian [81–88]	ins6Kb, c.68_69delAG c.211A>G* c.1082C>G c.2037delGinsCC c.3331_3334delCAAG 3261delGinsCC c.3403C>T c.5263dupC c.5266dupC*	- p.Glu23fs p.Arg71Gly* p.Ser361Ter p.Lys679fs p.Gln1111fs* - p.Gln1135Ter p.Glu1756Profs*4 p.Gln1756Profs	c.156_157insAlu* c.3869G>A c.4808delA c.5650_5659del c.5946_5946delT c.6405_6409delCTTAA c.6656C>G	p.Lys53AlafsTer9* p.Cys1290Tyr p.Asn1603fs p.Ile1884fsp.Ser1982Argfs p.Asn2135Lysfs p.Ser2219Ter p.Ser2219Ter
Chilean [89, 90]	c.68_69delAG c.211A>G c.1504_1507delTTAA* c.2486_2487delTT c.3331_3334delCAAG* 3759dupT* c.3817C>T*	p.Glu23fs p.Arg71Gly p.Leu502Serfs* p.Gly828_Phe829insTer p.Gln1111fs* p.Lys1254Terfs* p.Gln1273Ter*	c.145G>T* c.4740_4741insTG* c.5146_5149del4* c.6275_6276delT c.8987T>A* c.9382C<T*	p.Glu49Ter* p.Glu1581Trpfs* p.Tyr1716LysfsTer8* p.Leu2092Profs p.Leu2996* p.Arg3128Ter*
Colombian [91, 92]	c.114G>A c.211A>G* c.2808_2811delACAA* c.3331_3334delCAAG* c.4327C>T* c.5123C>A*	p.Lys38= p.Gln1111fs p.Ala1708Glu p.Arg1443Ter p.Ala1708Glu	ex1-14del c.1763_1766delATAA c.2808_2811del4* c.6024dupG	- p.Asn588Serfs p.Ala938Profs p.Gln2009Alafs
Costa Rican [93]	c.68_69del c.3403C>T c.5266dupC*	p.Glu23fs p.Gln1135Ter p.Gln1756Profs*	c.5279C>G c.5303_5304delTT c.5946_5946delT c.6174delT	p.Ser1760Ter p.Leu1768fs p.Ser1982Argfs p.Phe2058fs
Cuban [94]			c.3166C>T	p.Gln1056Ter
French Canadian [56, 95–98]	c.962 G> A c.1016dupA c.1961dupA c.2125_2126insA c.2834_2836delGTAinsC c.3649_3650insA c.3756_3759delGTCT c.4327C>T* c.3756_3759delGTCT c.5102_5103delTG	p.Trp321Ter p.Val340GlyfsTer p.Tyr655ValfsTer p.Phe709TyrfsTer p.Ser945ThrfsTer p.Ser1217TyrfsTer p.Ser1253ArgfsTer10 p.Arg1443Ter* p.Ser1253fs p.Leu1701GlnfsTer14	c.2588dupA c.2806_2809delAAAC c.3170_3174delAAAAG c.3545_3546delTT c.5857G>T* c.6275_6276delTT c.8537_8538delAG* c.9004 G>A	p.Asn863LysfsTer p.Ala938ProfsTer21 p.Lys1057fs p.Phe1182Ter p.Glu1953Ter* p.Leu2092ProfsTer p.Glu2846Glyfs* p.Glu3002Lys
Fillipino [99]	c.5335delC*	p.Gln1779fs*	c.4631delA*	p.Asn1544Thrfs*
Mexican [100, 101]	c.211A>G* c.212+1G>A c.548-?_4185 ?del exons 9–12del* ex8-9dup ex18-19del c.4327C>T*	p.Arg71Gly* - - - - - p.Arg1443Ter*		
Peruvian [102]	c.68_69delAG c.1961delA c.4327C>T*	p.Glu23fs p.Lys654fs p.Arg1443Ter*	c.2808_2811del4	p.Ala938Profs
Puerto Rican [103]			c.3922G>T*	p.Glu1308Ter*
**Asian countries by population**				
Ashkenazi Jewish [49, 104]	c.68_69delAG* c.5266dupC*	p.Glu23fs* p.Gln1756Profs*	c.5946_5946delT *	p.Ser1982Argfs*
Chinesse [75, 105–107]	c.66dup c.470_471delCT c.981_982delAT* c.1465G>T c.3181del* c.3257del c.3342_3345delAGAA c.5406+1_5406+3delGTA c.5470_5477delATTGGGCA* c.981_982delAT*	p.Glu23Argfs p.Ser157Terfs p.Cys328Terfs* p.Glu489Ter p.Glu1060_Ile1061insTer* p.Arg1085_Leu1086insTer p.Glu1115Terfs - p.Ile1824Aspfs* p.Cys328Terfs	c.1832C>A c.6591_6592delTG c.1963delC c.2808_2811delACAA c.3109C>T* c.7436_7805del370* c.9097_9098insA*	p.Ser611Ter p.Glu2198Asnfs p.Arg655Glufs p.Gln1037Ter* p.Asp2479GlyfsX46* p.T3033fs*
Indian [108, 109]	c.68_69delAG c.178_179delCA c.1016delA c.2864C>A c.3331_3334delCAAG c.4094delT c.5074+1G>A c.5137+1G>A c.5098delC c.5148T>G	p.Glu23fs p.Gln60Valfs p.Lys339Argfs p.Ser955Ter p.Gln1111Asnfs p.Leu1365Terfs - - p.Thr1700fs p.Tyr1716Ter	c.682-2A>G c.1907C>G c.4638dupT c.4779A>C c.5851_5854del4 c.8117A>G c.5946delT*	- p.Ser636Ter p.Asp1547Ter p.Glu1593Asp p.(Ser1951TrpfsTer11) p.Asn2706Ser p.Ser1982Argfs*
Iraqi [110]	c.68_69delAG	p.Glu23fs		
Japanese [111–113]	c.307T>A* c.188T>A * c.2389delGA c.2800C>T* c.3442delG	p.Leu63Ter* p.Glu797Thrfs p.Gln934Ter* p.Glu1148Argfs	c.1278delA c.2835C>A* c.5576_5579delTTAA* 5802delAATT* c.8504C>A c.9117G>A c.6952C>T c.8589dupA	p.Asn433Glnfs - p.lle1859Lysfs* - p.Ser2835Ter p.Pro3039Pro p.Arg2318Ter p.Ala2864Serfs
Korean [114]			c.1399A>T c.3744_3747delTGAG c.7480C>T*	p.Lys467Ter p.Ser1248Argfs p.Arg2494Ter
Pakistani [115–117]	c.3770_3771del* c.4065_4068del* c.4485-1G>A* c.4508C>A* c.5503C>T* exon 1-2 deletion*	p.Glu1257Glyfs* p.Asn1355Lysfs* - p.Ser1503* p.Arg1835* -		
Vietnamese [118]	c.66dupA c.5251C>T	p.Glu23Argfs p.Arg1751Ter	c.1399A>T c.3744_3747delTGAG c.4478_4481delAAAG c.7480C>T	p.Lys467Ter p.Ser1248Argfs p.Glu1493Valfs p.Arg2494Ter
**European countries by population**				
Austrian [119]	c.181T>G c.1687C>T* c.2676_2679delAAAG* c.3016_3019del4 c.5266dupC	p.Cys61Gly p.Gln563Ter* p.​Leu892_Lys893?fs* p.His1006Glnfs p.Gln1756Profs	c.8363G>A c.8754+1G>A c.3860del	p.Trp2788Ter - p.Asn1287fs
Belarusian [120]	c.181T>G c.5266dupC	p.Cys61Gly p.Gln1756Profs		
Belgian [121–123]	c.212+3A>G* c.2359dupG* c.2685_2686delAA* c.3661G>T*	- p.Glu787Glyfs* p.Pro897Lysfs* p.Glu1221Ter*	c.516+1G>A* c.6275_6276delTT* c.8904delC*	- p.Leu2092ProfsTer7*p.Val2969fs*
British [124, 125]	c.2681_2682delAA*(Scotland) c.4065_4068del4(NorthWest) exon 13 duplication(ins6kbEx13)*	p.Lys894Thrfs* p. Asn1355Lysfs10 -	c.6275_6276delTT* (Scotland) c.1929delG (North-West)	p.Leu2092ProfsTer7*p.Arg645fs
Cypriot [126, 127]			c.8755delG*	-
Czech [128, 129]	c.181T>G* c.5266dupC* c.3700_3704del5* exons 1–17 deletion* exons 5–14 deletion* c.7910_7914del5*	p.Cys61Gly* p.Gln1756Profs* p.​Cys61Gly - - p.Phe2638Ter*	c.7913_7917delTTCCT* c.8537_8538del2*	p.Phe2638Terfs* p.Glu2846GlyfsTer22*
Danish [130–132]	c.2475delC c.3319G>T c.3710delT exons 3-16 deletion* c.5266dupC	p.Asp825fs p.Glu1107Ter p.Ile1237Asnfs - p.Gln1756Profs	c.1310_1013del4 c.3847_3848delGT c.6373delA c.6486_6489del4	p.Lys437IlefsX22 p.Val1283Lysfs p.Thr2125Profs p.Lys2162AsnfsTer5
Finnish [133–137]	c.2684del2 c.3485delA c.3626delT c.4096+3A>G* c.4097-2A>G c.4327C>T* c.5251C>T	p.Ala895fs p.Asp1162Valfs p.Lys1208_Leu1209insTer - - p.Arg1443Ter* p.Arg1751Ter	c.8327T>G* c.771_775delTCAAA* c.7480C>T* c.9117+1G>A* c.9118-2A>G	p.Leu2776Ter* p.Asn257Lysfs* p.Arg2494Ter* - -
French [43, 64]	c.3481_3491del11* c.5030_5033delCTAA c.5128G>T	- Thr1677Ilefs2 p.Gly1710Ter	c.6644_6647delACTC	p.Tyr2215SerfsTer13
German [138–140]	c.181T>G c.2338C>T c.4065_4068del4 Exon 17 deletion* c.5266dupC*	p. Cys61Gly p.Gln780Ter p. Asn1355Lysfs10 - p.Gln1756Profs	c.1813dupA c.4478del4 c.9098dupA	p. Ile605Asnfs11 p.Glu1493Valfs p.Gln3034fs
Greek [141, 142]	c.5212G>A* c.5251C>T c.5266dupC* c.5467G>A 3782del10 4512insT	p.Gly1738Arg* p.Arg1751Ter p.Gln1756Profs* p.Ala1823Thr - -	c.4284dup*	p.Gln1429fs*
Hungarians [143]	c.68_69delAG c.181T>G* c.5266dupC*	p. Glu23fs p. Cys61Gly* p.Gln1756Profs*	c.9097dupA c.5946delT*	p.Thr3033Asnfs p.Ser1982Argfs*
Icelandic [53, 54]			c.771_775del5*	p.Asn257Lysfs*
Italian [67, 144, 145]	c.116G>A* c.3228_3229delAG*(Tuscany) c.3285delA*(Tuscany) c.1380dupA* (Tuscany) c.5062_5064del3* (Tuscany) c.4964_4982del19* (Calabria) c.5062_5064delGTT*(Nordeast)	p. Cys39Tyr* p.Gly1077Alafs* p.Lys1095Asnfs* p.Phe461Ilefs* p. Val1688del* p.Ser1655Tyrfs* p. Val1688del*	c.8537_8538delAG* (Sardinia)	p.Glu2846Glyfs*
Irish [146]	c.427G>T*	p.Glu143Ter*		
Lativian [147]	c.4035delA* c.5266dupC	p.Glu1346fs* p.Gln1756Profs		
Lithuanian [148, 149]	c.4035delA* c.5266dupC	p.Glu1346fs* p.Gln1756Profs		
Norwegian [150, 151]	c.1A>C c.697delGT * c.1016dupA* c.1556delA c.2351del7* c.3084del11* c.3178G>T* c.3228delAG* c.4745delA* c.5075-2A>C exons 1-13 deletion exon 13 duplication (ins6kbEx13)	p.Met1Val p.Val233Ter* p.Val340Glyfs* p.Lys519Argfs p.Ser784Trpfs* p.Asn1029Argfs* p.Glu1060Ter* p.Gly1077Alafs* p.Asp1582fs* - - - -	c.2808del4* c.3847delGT*	p.Ala938Profs* p. Val1283fs*
Polish [152]	c.181T>G* c.3700_3704del5 c.4035delA c.5266dupC*	p.Cys61Gly* p.Val1234fs p.Glu1346fs p.Gln1756Profs*		
Portuguese [87]			c.156_157insAlu*	p.Lys53AlafsTer9*
Russian [153]	c.68_69delAG c.181T>G* c.4034delA c.5266dupC*	p. Glu23fs p.Cys61Gly* p.Glu1346fs p.Gln1756Profs*		
Scandinavian [154]	c.2475delC c.5266dupC	p.Asp825fs p.Gln1756Profs		
Scottish/ Northern Irish [155]	c.2681_2682delAA	p.Lys894fs	c.6275_6276delTT	p.Leu2092Profs
Slovenian [156–158]	c.181T>G* c.181T>A c.1687C>T c.5266dupC	p.Cys61Gly* p.Cys61Ser p.Gln563Ter p.Gln1756Profs	c.6589delA* c.7806-2A>G*	p.Thr2197fs -
Spanish [159–161]	c.68_69delAG* c.211A>G* (Galicia) c.2900_2901dupCT c.3331_3334delCAAG c.5117G>A c.5123C>A c.470_471delCT c.5153-1G>A	p.Glu23Valfs* p.Arg71Gly* p.Pro968Leufs p.Gln1111Asnfs p.Gly1706Glu (p.Ala1708Glu) p.Leu156_Ser157insTer -	c.1813dupA c.2095C>T c.2808_2811del4 c.4030_4035delinsC c.5146_5149del4 c.6629_6630delAA c.9026_9030del5 c.9310_9311delAA	p.Ile605Asnfs p.Gln699 p.Ala938Profs p.Asn1344fs p.Tyr1716Lysfs p.Glu2210fs p.Tyr3009Serfs p.Lys3104ValfsTer
Swedish [69, 162–166]	c.1082_1092del11* c.1016dupA c.1687C>T* c.2475delC* c.3048_3052dupTGAGA* c.3171_3175dup* c.3626delT* (Northern) exon 13 duplication (ins6kbEx13)	p.Cys360_Ser361insTer* p. Val340LysfsX6 p.Gln563Ter* p.Asp825fs* p.Asn1018Metfs* p.Thr1189fs* p.Lys1208_Leu1209insTer -	c.4258delG	p.Asp1420fs

*Variant described in literature as a founder variant

The high rates of specific and recurrent/founder PVs have led to a scientifically valid initiative to offer limited genotyping platforms. Establishing a founder effect lies mainly in the reduction of costs. Cost remains a frequently mentioned barrier to genetic testing in some populations such as North Africa. If we manage to decrease costs, screening could be offered more widely and cover a larger number of women, and could offer the benefits of early or pre-symptomatic diagnosis. To date, the eligibility criteria for *BRCA1* and *BRCA2* genetic testing have been expanded and updated. Currently, both the National Comprehensive Cancer Network (NCCN) guidelines and European Society for Medical Oncology (ESMO) guidelines recommend genetic testing for *BRCA1* and *BRCA2* genes to women also with personal history of cancer (e.g. multiple primary BCs, if first diagnosis was ≤50 years old, early age of BC, co-occurrence of BC and OC, etc. [167]. The three Ashkenazi-Jewish founder PVs (c.66_67del, c.5266dup in *BRCA1*, and c.5946del in *BRCA2*) are offered as a variant testing panel for self-reported Ashkenazim. This approach is much less expensive than comprehensive gene sequencing. With advances in sequencing technologies, testing women for the panel of population-specific recurrent/founder variants may be a valuable advance for therapy decisions in BC and OC patients.

A panel of *BRCA1* and *BRCA2* variants, including close to 100 recurrent variants (HISPANEL), has been constructed with diverse variants from Hispanic women with BC from the USA, based on the information in manuscripts describing variants in *BRCA* genes from Latin American countries and data bases [168]. In Poland, Łukomska et *al*., recommend that all women with OC and first-degree female relatives should be tested for the panel of 18 founder variants in *BRCA1*, *BRCA2*, *PALB2*, and *RAD51C* [169]. In addition to the known founder deleterious variants in the Chinese population, Jiang et *al*., highlight that the recurrent PVs in BC patients could be taken as candidate genetic screening loci for a more efficient genetic screening of this population [170]. Studies from Egypt suggest wider screening of the founder PVs (c.68_69del and c.5266dupC in *BRCA1*) among high-risk families using the Pyrosequencing technique that could be an excellent platform for *BRCA* founder PVs analysis [171].

Identification of specific and recurrent/founder variants that could be included in a low-cost PV panel, used as a first line screening approach, would be useful in the North African region. Advance of a screening panel for specific/recurrent and founder PVs offers a simple, rapid, and affordable routine molecular diagnostic method for the clinical management of BC and/or OC patients and their unaffected family members [172]. The PVs panel can also include other recurrent worldwide PVs such as *BRCA1*c.1016dupA which has also been reported in other countries (Italy, Germany, and French-Canada), however allelotyping results indicated an independent origin of this PV. That would justify the inclusion of the *BRCA1*c.1016dupA into targeted variant screening panels in any population, irrespective of ethnic origin [44].

In addition, offering a Next Generation Sequencing (NGS)-based Multigene Panel Testing (MGPT) to BC and/or OC patients may significantly increase the detection rates of specific/recurrent and founder PVs in BC and/or OC predisposition genes beyond *BRCA. PALB2*, *CHEK2*, *ATM*, *MUTYH*, *MSH2*, and *RAD51C* have been shown to be the most frequently altered gene in BC and/or OC patients with negative test result for *BRCA* PVs [173–175]. Several recurrent or founder PVs have been described among some of these genes. For example, the c.444+1G>A PV was detected in 29% of Slovenian patients with *CHEK2* variant [176]. Two splicing PVs in *CHEK2* gene, an Eastern European founder variant c.444+1G>A and a novel c.319+2T>A, were recently discovered in Finnish BC patients [177]. The *PALB2* c.3113G>A PV was the most recurrent familial variant in Australian BC patients [178]. Two recurrent *PALB2* PVs (c.172_175delGA and c.509_510delGA) were observed in Polish BC and/or OC [179]. The c.159delT variant in *PALB2* gene was identified as a founder PV in the Finnish population [180]. These genetic alterations can be identified in the European.

and non-European population and could be tested with the specific/recurrent and founder variants which occur in the *BRCA* genes for a targeted genetic screening of the first level using NGS-based MGPT. The identification of additional specific/recurrent and founder variants will be important for promoting and potentially advancing to rapid founder-based *BRCA* and beyond *BRCA* point-of-care technology as a time- and cost-effective alternative [181].

## Data Availability

The datasets generated and analysed during the current study are available in the [Breast and ovarian cancer in North Africa (tables)] repository. Pub med (Open access): https://pubmed.ncbi.nlm.nih.gov/ Science Direct (Open access): https://www.sciencedirect.com/ Google Scholar (Open access): https://scholar.google.com/

## Abbreviations

ACMG-AMP: American College of Medical Genetics and Genomics-Association for Molecular Pathology
ASCO: American Society of Clinical Oncology
BC: Breast Cancer
BIC database: Breast Cancer Information Coredatabase
*BRCA*: BReastCAncer
HBOC: Hereditary breast and ovarian cancer
OC: Ovarian Cancer
PVs: Pathogenic Variants
UMD: Universal Mutation Database
VUS: Variant(s) of Unknown/uncertain Significance
